# Drug-Related Mutational Patterns in Hepatitis B Virus (HBV) Reverse Transcriptase Proteins From Iranian Treatment-Naïve Chronic HBV Patients

**DOI:** 10.5812/hepatmon.6712

**Published:** 2013-01-20

**Authors:** Mostafa Mahabadi, Mehdi Norouzi, Seyed Moayyed Alavian, Katayoon Samimirad, Talat Mokhtari Azad, Esmaeil Saberfar, Mahmood Mahmoodi, Fatemeh Ramezani, Hadi Karimzadeh, Reza Malekzadeh, Ghodrat Montazeri, Azim Nejatizadeh, Masood Ziaee, Farshid Abedi, Behrooz Ataei, Majid Yaran, Babak Sayad, Mohammad Hossein Somi, Gholamreza Sarizadeh, Ismaeil Sanei-Moghaddam, Fariborz Mansour-Ghanaei, Houshang Rafatpanah, Mohammad Amin Pourhosseingholi, Hossain Keyvani, Ebrahim Kalantari, Mehdi Saberifiroozi, Mohammad Ali Judaki, Shiva Ghamari, Maryam Daram, Zeinab Fazeli, Zahra Goodarzi, Abolfazl Khedive, Abdolvahab Moradi, Seyed Mohamad Jazayeri

**Affiliations:** 1 Hepatitis B Molecular Laboratory, Department of Virology, School of Public Health, Tehran University of Medical Sciences, Tehran, IR Iran; 2 Middle East Liver Diseases Center (MELD Centers), Tehran, IR Iran; 3 Hepatitis C Molecular Laboratory, Department of Virology, School of Public Health, Tehran University of Medical Sciences, Tehran, IR Iran; 4 The research and development department of Bayerpaul vaccine and pharmaceutical company, Tehran, IR Iran; 5 Department of Epidemiology, School of Public Health, Tehran University of Medical Sciences, Tehran, IR Iran; 6 Digestive Disease Research Center, Tehran University of Medical Sciences, Tehran, IR Iran; 7 Research Center for Molecular Medicine, Hormozgan University of Medical Sciences, Bandar Abbas, IR Iran; 8 Hepatitis Research Center, Department of Internal Medicine, Faculty of Medicine, Birjand University of Medical Sciences, Birjand, IR Iran; 9 Department of Infectious Disease, Mashhad University of Medical Sciences, Mashhad, IR Iran; 10 Infectious Diseases and Tropical Medicine Research Center, Isfahan University of Medical Sciences, Isfahan, IR Iran; 11 Kermanshah Liver Diseases and Hepatitis Research Center, Kermanshah, IR Iran; 12 Liver and Gastrointestinal Disease Research Center, Tabriz University of Medical Sciences, Tabriz, IR Iran; 13 Educational Region of Khouzestan Blood Transfusion Organization, Ahvaz, IR Iran; 14 Department of Gastroenterology, Zahedan University of Medical Sciences, Zahedan, IR Iran; 15 Gastrointestinal and Liver Diseases Research Center, Guilan University of Medical Sciences, Rasht, IR Iran; 16 Immunology Research Center, Mashhad University of Medical Sciences, Mashhad, IR Iran; 17 Gastroenterology and Liver Diseases Research Center, Shahid Beheshti University of Medical Sciences, Tehran, IR Iran; 18 Department of Virology, School of Medicine, Tehran University of Medical Sciences, Tehran, IR Iran; 19 Gholhack Medical Laboratory, Tehran, IR Iran; 20 Gastroenterohepatology Research Center, Shiraz University of Medical Sciences, Shiraz, IR Iran; 21 Department of Microbiology, School of Medicine, Golestan University of Medical Sciences, Gorgan, IR Iran

**Keywords:** Therapy, Drug-Resistance, Hepatitis B Virus, Iran

## Abstract

**Background:**

Immunomodulators and Nucleotide analogues have been used globally for the dealing of chronic hepatitis B virus (HBV) infection. However, the development of drug resistance is a major limitation to their long-term effectiveness.

**Objectives:**

The aim of this study was to characterize the hepatitis B virus reverse transcriptase (RT) protein variations among Iranian chronic HBV carriers who did not receive any antiviral treatments.

**Materials and Methods:**

Hepatitis B virus partial RT genes from 325 chronic in active carrier patients were amplified and directly sequenced. Nucleotide/amino acid substitutions were identified compared to the sequences obtained from the database.

**Results:**

All strains belonging to genotype D.365 amino-acid substitutions were found. Mutations related to lamivudine, adefovir, telbivudine, and entecavir occurred in (YMDD) 4% (n = 13), (SVQ) 17.23% (n = 56), (M204I/V + L180M) 2.45% (n = 8) and (M204I) 2.76% (n = 9) of patients, respectively.

**Conclusions:**

RT mutants do occur naturally and could be found in HBV carriers who have never received antiviral therapy. However, mutations related to drug resistance in Iranian treatment-naïve chronic HBV patients were found to be higher than other studies published formerly. Chronic HBV patients should be monitored closely prior the commencement of therapy to achieve the best regimen option.

## 1. Background

**Figure 1:**
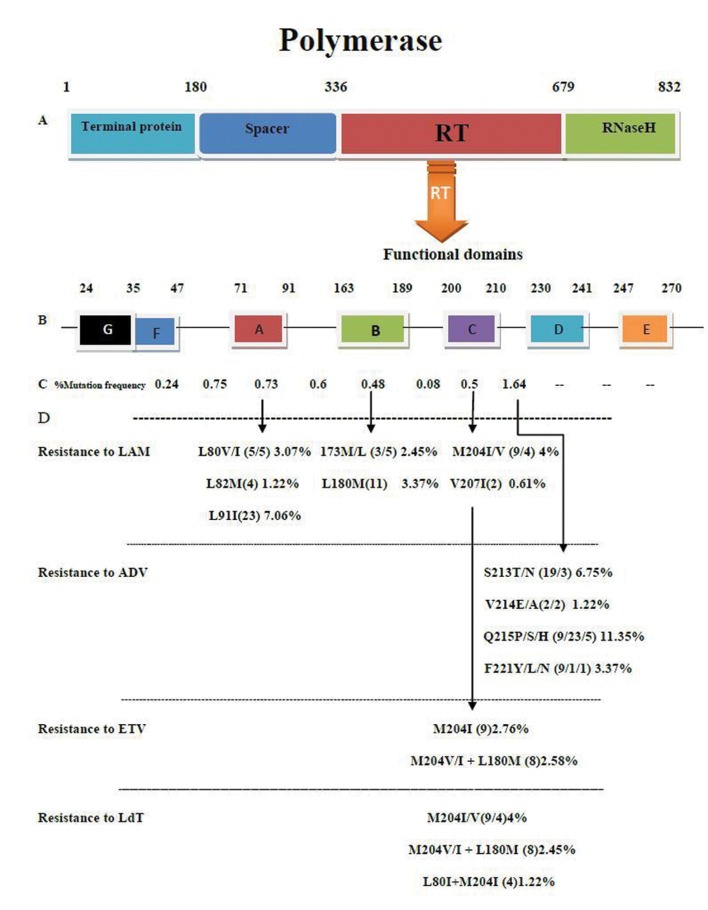
Schematic Figure Showing Polymerase Protein A) Four main domains include the RT region, B) RT region including domains and inter domains, C) Mutation frequency in the F domain to the C-D inter-domain, D) Individual drug-resistance mutation to LAM (Lamivudine), ADF (Adefovir), ETV (Entacavir), and LdT (Telbivudine).

Nearly 400 million individuals worldwide have been infected with chronic hepatitis B virus (HBV) (1), and of these, 75% are of Asian origin. These patients are at risk of developing progressive liver diseases including fibrosis, cirrhosis, or hepatocellular carcinoma and may require liver transplantation (2). Approved HBV therapies include immune modulators interferon alpha (IFN-a), peginterferonalpha (PegIFN-a2a), and nucleutide analogues (NAs) such as lamivudine, adefovir, entecavir, telbivudine, tenofovir, etc. Therapy reduces HBV DNA levels as much as possible, ideally less than the lower limit of detection of molecular assays, followed by biochemical remission, histological improvement, and prevention of complications. Although the initial effect of NA in suppressing HBV replication and reducing alanine aminotransferase activity is promising, the emergence of drug-resistant variants considerably reduces the benefit of therapy (3, 4). Drug resistance has been associated with the emergence of polymerase gene mutations that are localized within the RT region that consists of 6 functional domains (G, F, A, B, C, D and E) and 5 interdomains (F–A, A–B, B–C, C–D and D–E) (Figure 1, Tables 1 and 2). Among NAs, lamivudine and adefovir are the most commonly studied in the literature. Viral resistance emerges with both drugs, with frequencies as high as 30 and 80% after 4 years, respectively (5). Evidence now increasingly indicates that drug-related mutation does occur naturally and can be found in HBV carriers who have never received both therapies (6-8). The reported incidence of prevalence of YMDD mutations in treatment-naïve patients varies, ranging from 1 to 18% (9-12) in patients with chronic hepatitis B. The prevalence of HBV in Iran ranges from1.7 to 2.5% in the general population (2, 13) and both lamivudine and adefovir have widely been used in Iran.

**Table 1:** Mutations at AA Positions of HBV Reverse Transcriptase for Drug Resistance Reported With Known Phenotypic Data a

Type	Drugs	References
**L80V/I**	LAM	(7, 14)
**V84M**	ADV	(32)
**S85A**	ADV	(32)
**I169T**	ETV	(50)
**V173L**	LAM	(7, 14)
**L180M**	LAM, ETV, LDT	(7, 14)
**A181T/V**	LAM, ETV, LDT	(14, 25)
**T184G**	ETV	(7, 33)
**A194T**	TNF	(32, 34, 50)
**S202I/G**	ETV	(32, 50)
**M204I/V**	LAM,ETV, LDT	(34, 50)
**V214A**	ADV	(32, 51)
**Q215S**	ADV	(32, 51)
**I233V**	ADV	(32)
**N236T**	ADV	(32, 34, 51)
**P237H**	ADV	(7, 32)
**M250V**	ETV	(32, 51)

^a^These types of AA changes have been reported to be selected during NA therapy and associated with replication of hepatitis b virus in vitro

**Table 2:** Mutations at AA Positions of HBV Reverse Transcriptase Reported Genotypic Resistance for Drug Resistance

Type	Drugs	References
**S53N**	LAM	(7)
**T54N**	ADV	(7)
**L82M**	LAM	(31)
**I91L**	LAM	(7)
**Y126C**	ADV	(7)
**T128I/N**	LAM	(31)
**N139D/K/H**	LAM	(7)
**W153Q**	LAM	(7)
**F166L**	LAM	(7)
**V191I**	LAM.ADV	(31)
**A200V/P**	LAM	(7, 52)
**L217R**	ADV	(7, 52)
**V207I**	LAM	(31)
**S213T**	ADV	(7)
**S/C256G**	LAM,ETV	(7)
**L229G/V/W**	LAM	(25)
**I233V**	ADV	(7)
**N238K/D/S/T**	ADV	(7)
**Y245H**	ADV	(7)

## 2. Objectives

The aim of the present study was to explore the RT protein variations between Iranian HBV chronic carriers who had not received any type of HBV therapy.

## 3. Materials and Methods

### 3.1 Patients and Samples

Three hundred and twenty-five HBS Ag-positive patients who were referred to the Iranian Hepatitis Center (Tehran IR Iran) between April 2004 and April 2010, were enrolled in a cross-sectional study. All patients were given their informed consent and the study protocol was approved by the Iranian Hepatitis Network ethics committee. To cover all ethnic groups in the country, we studied 10 regions based on population and geographical zones. Exclusion criteria included hepatitis C virus, hepatitis D virus, and human immunodeficiency virus infection. All patients were interviewed and examined by gastroenterologists to evaluate the clinical findings and the results of the investigative workup(liver histology, ultrasonography, and laboratory tests such as serologic, biochemical, and virological tests) to determine the clinical status of the patient. All of the patients were chronic carriers of HBV. Next, 5 ml aliquots of whole blood samples were drawn from each participant. Serum was aseptically separated in the field by centrifugation at 4,000/rpm for 5 minutes and was stored at -20°C until tested. HBV serological markers including HBS Ag and anti-HBS were examined by ELISA kits manufactured by Organon Technika, Holland. 

### 3.2. DNA Extraction and Polymerase Chain Reaction and DNA Sequencing

 HBV DNA was extracted from 200 µl of sera using a Qiagen Mini Blood Kit (Qiagen, Hilden, Germany) according to the manufacturer’s instructions. DNA was stored at -20°C. First-round amplification was conducted by RTF CCT GCT GGT GGC TCC AGT TC as the sense primer and RTR CCA CAA TTC (K) TT GAC ATA CTT TCC A as the antisense primer.

 Second-round PCR was performed by RTNF GCA CAC GGA ATT CCG AGG ACT GGG GAC CCT G as the sense primer and RTNR GAC ACC AAG CTT GGT TAG GGT TTA AAT GTA TAC C as the antisense primer. The first-round PCR was run at 94°C for 4 minutes, followed by 35 cycles at 94°C for 30 seconds, 62°C for 35 seconds, and 72°C for 30 seconds, followed by 72°C for 10 minutes. A similar program was applied for the second-round PCR. Direct sequencing of PCR products was carried out (Perkin Elmer ABI-3130XL DNA Sequencer, Foster city, CA, USA) using 0.5 μl of appropriate primers RTF and RTR for the first cycle, an RTNF and RTNR for the second-round PCR products. 

### 3.3. Mutational Analysis

Amino acid variations within two hundred and thirty amino acid RT regions were compared with reference sequences obtained from different HBV genotype and sequences from Iranian isolates obtained from Gen Bank and NCBI. Comparing to the former, any amino acid changes defined as “variant”. With regards to the latter (Iranian database sequences), amino acid differences defined as “mutation”. The sequences were analyzed using Bio Edit Software version 7.0.5.3. Drug-related mutations in hepatitis B virus reverse transcriptase sequences within RT refereed and are listed in Tables 1 and 2. The amino acid mutation frequencies were obtained and by drug-resistance mutation found in individual RT domains (A, B, C and F) divided by the number of amino acid residues in that particular domain. The sequences sent to Gen bank under accession numbers from JX565097 to JX565421. 

### 3.4. HBV Genotyping and Phylogenetic Analysis

HBV genotyping was determined using NCBI Viral Genotyping Tool (http://www.ncbi.nlm.nih.gov/projects/genotyping/formpage.cgi). Phylogenetic analysis was conducted MEGA version 4. Briefly, sequences of HBV partial RT gene sequences (approximately 700-bp, 325 isolates in this study) were block aligned by the CLUSRAL X program and corrected visually, the Kimuratwo-parameter algorithm was used for genetic distance calculation. A phylogenetic tree was generated along with a different HBV genotype (A to H) sequences retrieved from the Gen Bank as reference genes by the neighbor-joining method, and bootstrapped-sampling and reconstruction was carried out 1000 × to confirm the reliability of the phylogenetic tree.

## 4. Results

### 4.1. Patient Characteristics

Three hundred and twenty five HBS Ag-positive chronic patients who were all were chronic carriers enrolled in this study, which were native residents of diverse regions of Iran, HBV DNA positive and treatment-naïve. Of total, 46.47% (151/325) were female and 53.53% (174/325) were male with a mean age of 36 years (SD ± 20). The mean ALT levels were 29 IU/L (SD ± 5) and 38% (123/325) were HBeAg positive. 

### 4.2. Genotypic and Phylogenetic Analysis

**Figure 2:**
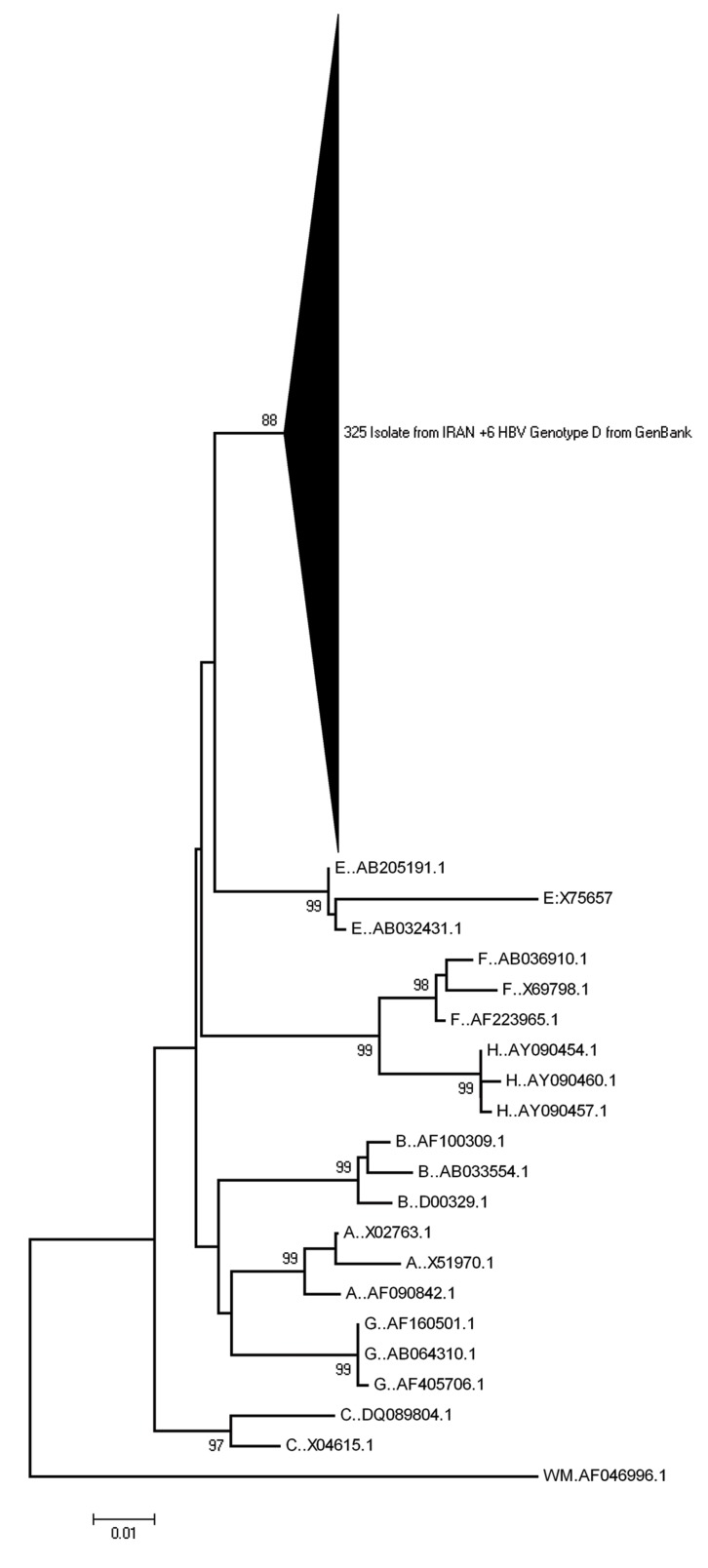
Neighbor joining phylogenetic analysis based on the alignment of the RT gene region (approximately 750-bp) of 325 HBV isolates from Iran as well as other HBV genotypes from Gen Bank as reference. Bootstrap values indicate 1000-foldreplicates. Due to clarity, the 325 isolates from Iran and 6 reference genes of HBV genotype D was collapsed. Woolly monkey HBV was used as the out-group sequence.

The phylogenetic tree was constructed using the alignment of HBVRT region of patients (325 isolates from this study) along with different HBV genotype (A to H) sequences retrieved from the Gen Bank as reference genes. The phylogenetic tree revealed that all Iranian isolates were branched with other genotype D of HBV reference isolates with a high boot strap value, 99%, 1000 × replicates (Figure 2).

### 4.3. Analysis of Amino Acid Substitutions

Compared to the reference genotypes D and according to the above-mentioned criteria for differentiation between variants and mutants, all patients contained at least one amino-acid mutation in the RT region. The total number of amino-acid mutations was 365, of which 14.5% (n = 53), 11.2% (n = 41), and 4.9% (n = 18) were found within the A, B and C domains, respectively. The mutation frequencies in the functional domains of the partial RTs were 0.73% (53/6825) in Domain A, 0.48% (41/8450) in Domain B, and 0.50% (18/3575) in Domain C. The average amino-acid substitution was 0.44%, indicating the probability of changes per position. In the mutational analysis of the RT inter domains, the substitutions and mutation frequencies were found in A-B0.60% (141/23400), B-C0.08% (3/3575), and C-D1.64% (107/6500) inter domains (Figure 1).

### 4.4. Lamivudine-Related Resistance Mutations 

Common and frequent lamivudine-related amino acid substitutions were found in this study including N53K 3.68% (12/325), L80V/I 3.07% (each 5/325), L82M1.22% (4/325) L91I 7.06% (23/325), T128I 1,53% (5/325), L180M3.37% (11/325), and M204I2.76%(9/325) M204V 1.23% (4/325) see Figure 1.

### 4.5. Adefovir-Related Resistance Mutation

RT sequence changes implicated in adefovir-resistance were detected, including S213T 5.83% (19/325), S213N 0.9% (3/325), V214E 0.61% (2/325), V214A 0.61% (2/325), Q215P 2.75% (9/325), Q215S 7.06% (23/325), Q215H1.53% (5/325), and F221Y 2.76% (9/325) of isolations that previously reported adefovir-resistance mutation (Figure 1). 

### 4.6. Other Drug-Related Resistance Mutation and Anti-Viral Cross-Resistance Mutations

Cross-resistance refers to the situation in which a decreased susceptibility to more than one antiviral drug is conferred by the same amino acid substitution or combination of amino acid substitutions. Cross-resistance mutations to lamivudineand telbivudinewere: M204I 2.76% (9/325), M204V 1.23% (4/325), M204V/I + L180M 2.45% (8/325), and L80I + M204I 1.23% (4/325). Cross-resistance mutations to lamivudine and entecavirwere: M204I 2.76% (9/325) and M204V/I + L180M 2.45% (8/325) (Figure 1).

## 5. Discussion

Hepatitis B virus DNA polymerase primary resistance mutations in treatment-naïve patients have been reported in variable frequencies and with unclear clinical significance in different studies (9-12). The aim of this study was to characterize the mutational patterns of RT protein in chronic HBV carriers. All 325 patients included in this study were treatment naïve. All had been infected with genotype D. The results showed that viral mutations related to lamivudine, adefovir ,telbivudine, and entecavir resistance were discovered in patients who had never been treated with these drugs, with various mutation frequencies ranging between 1.23% (for M204V) and 7.06% (for L91I). However in the present study, the molecular cloning was not performed, and the results of HBV DNA polymerase mutations were obtained from direct sequencing techniques; therefore, the actual number of mutations might be underestimated (14). Lamivudine was the first effective oral HBV-replication suppressive agent to enter widespread clinical use; however, its use had been challenged with the emergence of resistant mutations, which increased from 23% to 80% following the first and fourth years, respectively (15, 16). The most commonly described mutations are the substitution of valine or isoleucine for methionine at residue 204 (rtM204I/V). These YMDD motif mutations are necessary and sufficient to confer a high level of lamivudine resistance. For this specific mutation, the rtL180M is the main compensatory change. Other compensatory mutations include the rtV173L and rtL80I changes (17, 18). The rtA181T change has been reported to occur in the absence of rtM204I/V and is considered a primary resistance mutation (19, 20). In this study we found that L180M 3.37% (11), M204I 2.76% (9), and M204V 1.23% (4) were more common than had been reported previously in Iran (21, 22). Our results fall between the extremes reported by other studies around the world (7-12, 23). A Adefovir dipivoxil has been shown to be effective against both wild-type and lamivudine-resistant HBV both in vitro and in vivo (18-20). Compared to the lamivudine, resistance to ADV usually occurs less frequently and takes longer to emerge. Adefovir-resistant HBV mutations have not been detected in patients up to 48 weeks into therapy and increase to 3%, 11%, 18%, and 29% following 2, 3, 4, and 5 years of therapy, respectively (22, 24). HBV genotype D is associated with an increased risk of adefovir resistance (11, 25). Adefovir resistance has been associated with a primary mutation in the D domain at rtN236T and rtA181T/Vin the B domain. In addition, a number of other mutations have been detected in our study and were clustered into three distinct regions of the RT: the D and A domains (rtP237H, rtN238T/D, rtV84M, and rtS85A) and the C-D interdomain (rtS213T/N, rtV214A, and rtQ215S) (26-31). The latter finding had a mutation frequency of 1.64% and was higher than the other domains studied (Figure 1). These mutations may be regarded as secondary resistance mutations, as they have been associated only with very low-level resistance in vitro (22, 26). These secondary mutations have also been detected in the absence of rtN236T (both alone and in combination) in patients who have either not responded to or have had a virological breakthrough during adefovir treatment (32-34). The high number of adefovir-related mutation in this region may be indicative of an increased risk of adefovir resistance in genotype D-infected patients if they receive this drug (11, 25). Preliminary evidence indicates that the primary lamivudine-resistance mutation rtM204V/I seems to be a prerequisite for the development of entecavir and telbivudine resistance (35). Telbivudine and entecavir-related RT mutations were also found in this study. M204I/V and M204V/I/L180M are related to entecavir resistance (36-39). Recently, the GLOBE-Trial demonstrated that in vitro telbivudine resistance is conferred by either rtM204I or rtM204V+rtL180M and L80I/M204I but not by the rtM204V mutation alone, while in vivo telbivudine resistance is almost exclusively due to the presence of M204I. The reasons for the presence of such mutations in treatment-naïve chronic patients are not clear. Polymerase is an essential structural protein with a high error rate resembling RNA viruses. As a quasi-species in the hepatitis B virus pool, a cocktail of variants could be found in a single patient even in each body compartment with distinguished sequences from the master sequence (wild type). Due to a relatively high mutation rate of HBV (a rate between cellular and RNA virus replication), a tremendous number of mutations are produced and accumulate as spectra within the host. Some of these variants are not necessarily infectious (40). Furthermore, intensive investigation on RNA viruses (with a mutation rate close to HBV) shows that even in the absence of selective pressure, a minority of sequence variants exist as “memory,” which is a property of the population as a whole contained within the components of the mutant spectra (41, 42). This memory provides an adaptive advantage to the viral populations (43) and is eliminated when severe bottlenecks occur (44, 45). They gain fitness and can constitute a reservoir of new variants that display increased replication capacity in the environment together with the dominant genome populations (46). Together, these preexisting variants, which have less replicative ability than wild-type variants, can be reorganized in the presence of drugs in vivo and in vitro (like LAM, ADV, etc.) and encompass the wild-type variants, which has led to the drug-resistance phenomenon as it has shown for HCV (47) and HIV (47, 48) viruses. From a clinical perspective, the treatment-naïve patients in our study may have been infected with strains from other patients who had been treated with the corresponding nucleotide analogues. In this regard, differences in the distribution of HLA antigens or other immune genes across diverse geographical areas probably contributed to the selection of amino-acid variations. However, primary data need to be accumulated to prove this hypothesis (49). In conclusion, the clinical impact of pretreatment mutations on the efficacy of antiviral therapy should be better characterized. For the desired benefits in a cost-effective manner in the management of chronic HBV patients, genotypic and phenotypic screening before the decision to pursue antiviral therapy (as a genotypic approach) seems to be advisable.

## References

[1] Kose S, Turken M, Cavdar G, Akkoclu G (2010). The Effectiveness of Nucleoside Analogues in Chronic Hepatitis B Patients Unresponsive to Interferon Therapy: Our Clinical Trials for One Year.. Hepat Mon..

[2] Alavian SM, Fallahian F, Lankarani KB (2007). The changing epidemiology of viral hepatitis B in Iran.. J Gastrointestin Liver Dis..

[3] Marcellin P, Asselah T (2005). Resistance to adefovir: a new challenge in the treatment of chronic hepatitis B.. J Hepatol..

[4] Locarnini S, Qi X, Arterburn S, Snow A, Brosgart CL, Currie G (2005). Incidence and predictors of emergence of adefovir resistant HBV during four years of adefovir dipivoxil (ADV) therapy for patients with chronic hepatitis B (CHB).. J Hepatol..

[5] Deny P, Zoulim F (2010). Hepatitis B virus: from diagnosis to treatment.. Pathol Biol (Paris)..

[6] Han Y, Huang LH, Liu CM, Yang S, Li J, Lin ZM (2009). Characterization of hepatitis B virus reverse transcriptase sequences in Chinese treatment naive patients.. J Gastroenterol Hepatol..

[7] Liu BM, Li T, Xu J, Li XG, Dong JP, Yan P (2010). Characterization of potential antiviral resistance mutations in hepatitis B virus reverse transcriptase sequences in treatment-naive Chinese patients.. Antiviral Res..

[8] Nguyen MH, Garcia RT, Trinh HN, Nguyen HA, Nguyen KK, Nguyen LH (2009). Prevalence of hepatitis B virus DNA polymerase mutations in treatment-naive patients with chronic hepatitis B.. Aliment Pharmacol Ther..

[9] Akarsu M, Sengonul A, Tankurt E, Sayiner AA, Topalak O, Akpinar H (2006). YMDD motif variants in inactive hepatitis B carriers detected by Inno-Lipa HBV DR assay.. J Gastroenterol Hepatol..

[10] Feeney E, Fanning LJ, Horgan M (2007). Baseline genotypic resistance in untreated hepatitis B virus infection.. Gastroenterology..

[11] Fung SK, Chae HB, Fontana RJ, Conjeevaram H, Marrero J, Oberhelman K (2006). Virologic response and resistance to adefovir in patients with chronic hepatitis B.. J Hepatol..

[12] Margeridon-Thermet S, Shulman NS, Ahmed A, Shahriar R, Liu T, Wang C (2009). Ultra-deep pyrosequencing of hepatitis B virus quasispecies from nucleoside and nucleotide reverse-transcriptase inhibitor (NRTI)-treated patients and NRTI-naive patients.. J Infect Dis..

[13] Merrill RM, Hunter BD (2011). Seroprevalence of markers for hepatitis B viral infection.. Int J Infect Dis..

[14] Lok AS, Zoulim F, Locarnini S, Bartholomeusz A, Ghany MG, Pawlotsky JM (2007). Antiviral drug-resistant HBV: standardization of nomenclature and assays and recommendations for management.. Hepatology..

[15] Lai CL, Dienstag J, Schiff E, Leung NW, Atkins M, Hunt C (2003). Prevalence and clinical correlates of YMDD variants during lamivudine therapy for patients with chronic hepatitis B.. Clin Infect Dis..

[16] Leung NW, Lai CL, Chang TT, Guan R, Lee CM, Ng KY (2001). Extended lamivudine treatment in patients with chronic hepatitis B enhances hepatitis B e antigen seroconversion rates: results after 3 years of therapy.. Hepatology..

[17] Delaney WEt, Yang H, Westland CE, Das K, Arnold E, Gibbs CS (2003). The hepatitis B virus polymerase mutation rtV173L is selected during lamivudine therapy and enhances viral replication in vitro.. J Virol..

[18] Ogata N, Fujii K, Takigawa S, Nomoto M, Ichida T, Asakura H (1999). Novel patterns of amino acid mutations in the hepatitis B virus polymerase in association with resistance to lamivudine therapy in japanese patients with chronic hepatitis B.. J Med Virol..

[19] Ono SK, Kato N, Shiratori Y, Kato J, Goto T, Schinazi RF (2001). The polymerase L528M mutation cooperates with nucleotide binding-site mutations, increasing hepatitis B virus replication and drug resistance.. J Clin Invest..

[20] Yeh CT, Chien RN, Chu CM, Liaw YF (2000). Clearance of the original hepatitis B virus YMDD-motif mutants with emergence of distinct lamivudine-resistant mutants during prolonged lamivudine therapy.. Hepatology..

[21] Amini-Bavil-Olyaee S, Hosseini SY, Sabahi F, Alavian SM (2008). Hepatitis B virus (HBV) genotype and YMDD motif mutation profile among patients infected with HBV and untreated with lamivudine.. Int J Infect Dis..

[22] Ramezani A, Velayati AA, Roshan MR, Gachkar L, Banifazl M, Keyvani H (2008). Rate of YMDD motif mutants in lamivudine-untreated Iranian patients with chronic hepatitis B virus infection.. Int J Infect Dis..

[23] Briones C, de Vicente A, Molina-Paris C, Domingo E (2006). Minority memory genomes can influence the evolution of HIV-1 quasispecies in vivo.. Gene..

[24] Hadziyannis SJ, Tassopoulos NC, Heathcote EJ, Chang TT, Kitis G, Rizzetto M (2005). Long-term therapy with adefovir dipivoxil for HBeAg-negative chronic hepatitis B.. N Engl J Med..

[25] Locarnini S (2008). Primary resistance, multidrug resistance, and cross-resistance pathways in HBV as a consequence of treatment failure.. Hepatol Int..

[26] Arrese E, Basaras M, Blanco S, Ruiz P, Cisterna R (2010). Monitoring of therapy in patients with chronic hepatitis B virus.. Eur J Gastroenterol Hepatol..

[27] Curtis M, Zhu Y, Borroto-Esoda K (2007). Hepatitis B virus containing the I233V mutation in the polymerase reverse-transcriptase domain remains sensitive to inhibition by adefovir.. J Infect Dis..

[28] Kim DY, Ahn SH, Chang HY, Shim HY, Heo J, Cho M (2008). 563 Hepatitis B Virus Quasispecies in the Polymerase Gene in Treatment-Naive Chronic Hepatitis B Patients.. J Hepatol..

[29] Shaw T, Bartholomeusz A, Locarnini S (2006). HBV drug resistance: mechanisms, detection and interpretation.. J Hepatol..

[30] Sheldon J, Ramos B, Garcia-Samaniego J, Rios P, Bartholomeusz A, Romero M (2007). Selection of hepatitis B virus (HBV) vaccine escape mutants in HBV-infected and HBV/HIV-coinfected patients failing antiretroviral drugs with anti-HBV activity.. J Acquir Immune Defic Syndr..

[31] Sheldon J, Rodes B, Zoulim F, Bartholomeusz A, Soriano V (2006). Mutations affecting the replication capacity of the hepatitis B virus.. J Viral Hepat..

[32] Ghany M, Liang TJ (2007). Drug targets and molecular mechanisms of drug resistance in chronic hepatitis B.. Gastroenterology..

[33] Reijnders JG, Leemans WF, Hansen BE, Pas SD, de Man RA, Schutten M (2009). On-treatment monitoring of adefovir therapy in chronic hepatitis B: virologic response can be assessed at 24 weeks.. J Viral Hepat..

[34] Seifer M, Patty A, Serra I, Li B, Standring DN (2009). Telbivudine, a nucleoside analog inhibitor of HBV polymerase, has a different in vitro cross-resistance profile than the nucleotide analog inhibitors adefovir and tenofovir.. Antiviral Res..

[35] Sayan M (2010). Molecular diagnosis of entecavir resistance.. Hepat Mon..

[36] Alavian SM, Hajarizadeh B, Ahmadzad-Asl M, Kabir A, Lankarani KB (2008). Hepatitis B Virus Infection in Iran: A Systematic Review.. Hepat Mon..

[37] Hajiani E, Hashemi SJ, Masjedizadeh AR (2009). Seroepidemiology of Hepatitis B Virus Infection in Khuzestan Province, Southwest of Iran.. Hepat Mon..

[38] Locarnini S, Bowden S (2010). Drug resistance in antiviral therapy.. Clin Liver Dis..

[39] Nokhodian Z, Kassaian N, Ataei B, Javadi AA, shoaei P, Farajzadegan Z (2009). Hepatitis B Markers in Isfahan, Central Iran: A Population-Based Study.. Hepat Mon..

[40] Grande-Perez A, Lazaro E, Lowenstein P, Domingo E, Manrubia SC (2005). Suppression of viral infectivity through lethal defection.. Proc Natl Acad Sci U S A..

[41] Arias A, Ruiz-Jarabo CM, Escarmis C, Domingo E (2004). Fitness increase of memory genomes in a viral quasispecies.. J Mol Biol..

[42] Ruiz-Jarabo CM, Arias A, Baranowski E, Escarmis C, Domingo E (2000). Memory in viral quasispecies.. J Virol..

[43] Domingo E (2010). Mechanisms of viral emergence.. Vet Res..

[44] Domingo E, Escarmis C, Lazaro E, Manrubia SC (2005). Quasispecies dynamics and RNA virus extinction.. Virus Res..

[45] Ruiz-Jarabo CM, Miller E, Gomez-Mariano G, Domingo E (2003). Synchronous loss of quasispecies memory in parallel viral lineages: a deterministic feature of viral quasispecies.. J Mol Biol..

[46] Wyatt CA, Andrus L, Brotman B, Huang F, Lee DH, Prince AM (1998). Immunity in chimpanzees chronically infected with hepatitis C virus: role of minor quasispecies in reinfection.. J Virol..

[47] Briones C, Domingo E (2008). Minority report: hidden memory genomes in HIV-1 quasispecies and possible clinical implications.. AIDS Rev..

[48] Briones C, Domingo E, Molina-Paris C (2003). Memory in retroviral quasispecies: experimental evidence and theoretical model for human immunodeficiency virus.. J Mol Biol..

[49] Jazayeri SM, Carman WF (2005). Virus Escape CTL or B Cell Epitopes?. Hepat Mon..

[50] Bartholomeusz A, Locarnini SA (2006). Antiviral drug resistance: clinical consequences and molecular aspects.. Semin Liver Dis..

[51] Locarnini S, Mason WS (2006). Cellular and virological mechanisms of HBV drug resistance.. J Hepatol..

[52] Cuestas ML, Rivero CW, Minassian ML, Castillo AI, Gentile EA, Trinks J (2010). Naturally occurring hepatitis B virus (HBV) variants with primary resistance to antiviral therapy and S-mutants with potential primary resistance to adefovir in Argentina.. Antiviral Res..

